# Domain-Adaptive Graph Attention Semi-Supervised Network for Temperature-Resilient SHM of Composite Plates

**DOI:** 10.3390/s25226847

**Published:** 2025-11-09

**Authors:** Nima Rezazadeh, Alessandro De Luca, Donato Perfetto, Giuseppe Lamanna, Fawaz Annaz, Mario De Oliveira

**Affiliations:** 1 School of Architecture, Built Environment, Computing and Engineering, Birmingham City University, Birmingham B4 7XG, UK; fawaz.annaz@bcu.ac.uk (F.A.); mario.deoliveira@bcu.ac.uk (M.D.O.); 2 Department of Engineering, University of Campania “Luigi Vanvitelli”, 81031 Aversa, Italy; alessandro.deluca@unicampania.it (A.D.L.); donato.perfetto@unicampania.it (D.P.); giuseppe.lamanna@unicampania.it (G.L.)

**Keywords:** structural health monitoring (SHM), composite materials, graph attention networks (GATs), domain adaptation (DA), temperature variability, explainability

## Abstract

This study introduces GAT-CAMDA, a novel framework for the structural health monitoring (SHM) of composite materials under temperature-induced variability, leveraging the powerful feature extraction capabilities of Graph Attention Networks (GATs) and advanced domain adaptation (DA) techniques. By combining Maximum Mean Discrepancy (MMD) and Correlation Alignment (CORAL) losses with a domain-discriminative adversarial model, the framework achieves scalable alignment of feature distributions across temperature domains, ensuring robust damage detection. A simple yet at the same time efficient data augmentation process extrapolates damage behaviour across unmeasured temperature conditions, addressing the scarcity of damaged-state observations. Hyperparameter optimisation via Optuna not only identifies the optimal settings to enhance model performance, achieving a classification accuracy of 95.83% on a benchmark dataset, but also illustrates hyperparameter significance for explainability. Additionally, the GAT architecture’s attention demonstrates the importance of various sensors, enhancing transparency and reliability in damage detection. The dual use of Optuna serves to refine model accuracy and elucidate parameter impacts, while GAT-CAMDA represents a significant advancement in SHM, enabling precise, interpretable, and scalable diagnostics across complex operational environments.

## 1. Introduction

Ultrasonic-guided waves (UGWs), particularly Lamb waves, have become fundamental in SHM due to their ability to propagate over long distances with minimal attenuation, making them highly effective for inspecting large structures such as wind turbine blades and aerospace components. Their high sensitivity to both surface and internal defects facilitates robust damage detection when interacting with structural anomalies that alter propagation characteristics [1,2,3]. Piezoelectric sensors, pivotal in generating and detecting Lamb waves, offer a scalable and cost-effective SHM solution, providing widespread monitoring capabilities [4,5]. However, the multimodal and dispersive nature of Lamb waves introduces significant challenges in interpreting signals and accurately localising damage. These difficulties are compounded by environmental and operational variabilities (EOVs) such as temperature, humidity, and mechanical stress, which distort wave propagation and impair system reliability [2,6]. In composite materials such as Carbon Fibre-Reinforced Polymer (CFRP), the heterogeneity and anisotropy of the material further necessitate specialised techniques to manage failure modes, including delamination and matrix cracking [7,8,9,10].

### 1.1. Temperature-Induced Variability and Conventional Compensation Methods

Among the various EOVs, temperature changes can influence guided wave propagation by affecting wave speed, phase, and amplitude, potentially masking or mimicking damage signatures [11,12,13]. Numerous compensation strategies have been proposed to mitigate these effects. Scheerer and Lager [14] evaluated three temperature compensation techniques (Best Baseline, Signal Interpolation, and Frequency Shift), concluding that Frequency Shift was effective under moderate temperature variations (≤5 °C). In addition, statistical modelling approaches have been applied to tackle temperature-induced variability. For example, Silva et al. [15] used auto-regressive models with cubic spline extrapolation to track damage progression under temperature fluctuations, although the approach required extensive baseline data and thus faced scalability limitations. Ren et al. [16] adopted Gaussian Process Regression with Monte Carlo sampling to quantify uncertainty in damage detection, demanding large datasets that may not be practical for real-time SHM. Complementing these statistical methods, semi-analytical and finite element (FE) modelling also feature prominently. Ren et al. [17] explored temperature compensation in composite structures through a semi-analytical FE model, validating their findings experimentally but relying on well-defined material and geometric properties. Similarly, Perfetto et al. [18] studied guided wave propagation under thermal loads using FE modelling, deriving time compensation factors that nonetheless require extensive experimental corroboration.

### 1.2. Data-Driven Methods for Temperature Compensation

Given the limitations of purely model-based and statistical strategies, data-driven methods are increasingly used to compensate for temperature effects [19]. Ferreira et al. [20] proposed a Bayesian framework that integrates FE model updating with neural networks to produce synthetic datasets for temperature compensation, reducing reliance on physical experiments but introducing potential inaccuracies when dealing with poorly characterised conditions. Giannakeas et al. [21] presented an up-scaling methodology using Bayesian regression to extend temperature compensation factors across structural scales, thus improving damage detection reliability, though at the expense of comprehensive validation efforts. Meanwhile, Cheng et al. [22] combined fibre optic sensors with piezoelectric transducers in a hybrid system for guided wave monitoring, although the sensitivity adjustments required for fibre optic sensors add complexity to the system.

GATs have garnered particular interest for capturing intricate spatial-temporal dependencies in graph-structured sensor data [23,24]. Niu et al. [25], for instance, leveraged attention mechanisms within GATs to handle incomplete SHM datasets by accounting for spatial-temporal relationships among sensors. Zhao et al. [26] further demonstrated a virtual sensor for bearing load prediction using heterogeneous temporal graph neural networks that explicitly modelled sensor signal dependencies and outperformed standard convolutional neural networks in load estimation tasks.

### 1.3. Transfer Learning for Mitigating EOVs in SHM

While data-driven models can capture complex wave behaviours, they often depend on large, high-quality labelled datasets that may not be available across different environmental regimes. Transfer learning (TL) therefore offers a promising solution, focusing on translating knowledge obtained in one domain to enhance performance in another [27]. Among TL methods, adversarial approaches such as Domain-Adversarial Neural Networks (DANNs) operate by minimising discrepancies between source and target distributions, thereby generating domain-invariant features. Ozdagli and Koutsoukos [28] demonstrated this technique in SHM, achieving improved cross-domain damage detection. Other feature-based alignment methods have also emerged: Zhuojun et al. [29] used MMD to reconcile simulated and experimental Lamb wave data for precise damage localisation, and Wang et al. [30] combined MMD with techniques including Variational Mode Decomposition and Transfer Component Analysis (TCA) to boost damage detection performance. Although many of these studies investigated TL under EOVs such as variations in loading or operating conditions, they rarely addressed temperature explicitly, which remains a critical and underexplored factor influencing guided wave propagation and model generalisation.

CORAL offers another feature-alignment strategy, albeit less commonly applied to UGW-based SHM. Poole et al. [31] employed CORAL in a partial DA setting, illustrating the benefit of aligning source and target covariance matrices for population-based SHM. By normalising datasets to account for anticipated EOVs, CORAL mitigates negative transfer and class imbalance, thereby improving damage localisation even with limited target-domain data. These advances collectively illustrate how TL can address data scarcity and ensure robust performance under varying environmental or operational conditions, making it an attractive approach for temperature compensation.

### 1.4. The Need for Explainability in ML-Based SHM

Despite these advances in data-driven and TL approaches, the opaque nature of many ML algorithms can hinder adoption in critical SHM tasks, where trust, interpretability, and actionable insights are essential. Explainable AI methods, including SHapley Additive exPlanations (SHAP) [32] and Local Interpretable Model-agnostic Explanations (LIME) [33], have been proposed to tackle the “black box” problem. As highlighted by Schnur et al. [34], neural networks are widely employed in SHM applications; however, their lack of interpretability limits their suitability for safety-critical contexts. Consequently, machine learning outcomes must be physically interpretable to facilitate their deployment in such domains.

To this end, Salih et al. [35] integrated SHAP and LIME into SHM workflows, with SHAP quantifying feature contributions and LIME providing localised signal interpretations. Tiwari et al. [36] harnessed SHAP in an ensemble approach for estimating shear strength in reinforced concrete, highlighting its utility in identifying sensor contributions. Azad and Kim [37] developed an explainable Vision Transformer for polymer composite diagnostics, employing attention mechanisms to highlight crucial decision regions, although at a substantial computational cost.

### 1.5. Main Contributions

This research addresses key limitations in the SHM of composite plates under temperature-induced EOVs through a novel framework, GAT-CAMDA. The main contributions are as follows:

- Combines multiple temperature domains into a single target domain, improving generalisation and reflecting real-world variability for greater robustness and practicality.
- Integrates CORAL and MMD losses to align feature distributions across temperatures, explores CORAL’s effectiveness in SHM, and employs GATs to capture complex spatial-temporal dependencies in UGW data for accurate damage detection.
- Uses GAT attention weights to visualise and quantify sensor contributions, enhancing model transparency and providing valuable insights into sensor importance for both theoretical and practical SHM applications.
- Employs Optuna [37], an automated hyperparameter optimisation framework, to systematically fine-tune model parameters within the semi-supervised process, thereby enhancing robustness and generalisability across temperature domains without requiring labelled target data for the DA stage [37].

The novelty of this work lies in its ability to generalise across diverse temperature conditions by merging multiple temperature domains into a unified framework. In contrast to previous studies restricted to narrow or isolated settings, this approach more closely represents real-world environmental variabilities. Furthermore, the thorough investigation of CORAL loss, an underexplored method in SHM applications, combined with the advanced feature extraction capabilities of GATs, marks a substantial step forward in aligning feature distributions and extracting discriminative patterns. By incorporating explainability through attention-weight visualisation and leveraging semi-supervised optimisation with Optuna, the proposed framework not only enhances damage detection accuracy but also delivers a transparent and scalable solution, setting a higher benchmark in SHM under complex environmental conditions.

The remainder of this paper is structured as follows: Section 2 presents the proposed framework, detailing its methodological advancements along with the implemented data synthesising technique. Section 3 outlines the case study, including the dataset and experimental setup. Section 4 discusses the results and evaluates the framework’s performance, and Section 5 concludes with key findings and future directions.

## 2. Materials and Methods

### 2.1. Overview of the Proposed Framework

GAT-CAMDA is a semi-supervised deep learning framework designed to address the challenge of SHM across different environmental conditions subjected to data scarcity. This pipeline comprises multiple stages, beginning with feature extraction using GATs, followed by DA through DANN enhanced with MMD and CORAL losses, and concluding with damage detection in the target domain system. To address data scarcity, damaged responses at target temperatures were estimated by leveraging temperature-dependent trends in healthy signals. The objective function in the optimisation procedure (Optuna) was to maximise the classification accuracy on the validation set of the target domain. To improve the explainability of the framework, the contributions of various hyperparameters and the significance of individual sensors were calculated and visualised.

In this study, the source domain refers to the data collected from a structural system under baseline environmental conditions (e.g., at a reference temperature), where labelled observations of both healthy and damaged states are available. The target domain comprises the data from the same structural system but under different environmental conditions (e.g., at various temperatures), where labelled damaged observations are scarce or unavailable. The fundamental challenge is to transfer knowledge learned from the source domain to accurately detect damage in the target domain, despite the distribution shift caused by EOVs.

**Figure 1:**
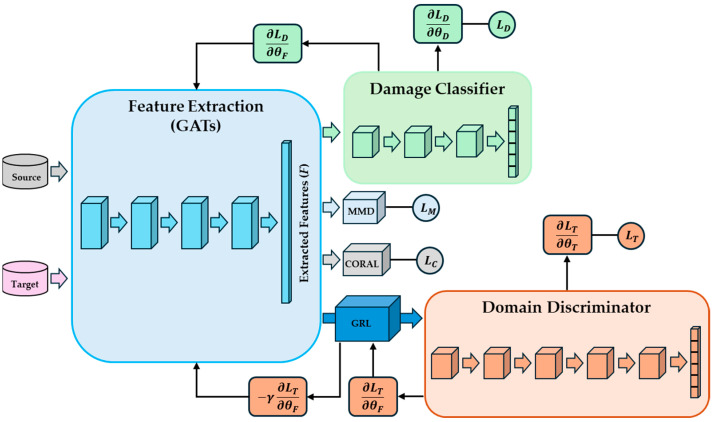
A schematic of GAT-CAMDA.

Figure 1 displays a schematic of the feature extraction and DA stages of GAT-CAMDA on the assumption that there are four GATs layers.

In the following parts each of these blocks are elaborated.

### 2.2. Graph Attention Networks

GATs were implemented as the feature extractor to capture both local and global relational structures within the input data. While conventional feature extraction methods excel at identifying localised patterns, they often struggle to comprehensively represent the complex interdependencies present in certain datasets more specifically, on the assumption that there are multi-sensor readings. GATs address this challenge by employing attention mechanisms that dynamically weight the importance of connections based on the significance of neighbouring features [38], thereby enabling a richer and more contextually informed representation. The process of a GAT can be summarised as follows [23]:

1. Feature transformation: Each node’s input feature vectors $x_{i}$ and $x_{j}$ undergo a shared linear transformation:

(1) $$x_{i}^{\prime }=Wx_{i},\;x_{j}^{\prime }=Wx_{j}$$

where W is a learnable weight matrix; the transformed feature vectors $x_{i}^{\prime }$ and $x_{j}^{\prime }$ are then used in subsequent steps.

2. Computation of importance scores: A self-attention mechanism computes unnormalised importance scores $e_{ij}$, quantifying the relevance of the j-th node’s features to the i-th node:

(2) $$e_{ij}=a\left(x_{i}^{\prime },\;x_{j}^{\prime }\right)$$

where a is a learned vector that parameterises the attention score.

3. Normalisation of attention coefficients: The scores $e_{ij}$ are normalised using the softmax function to produce attention coefficients $\alpha_{ij}$:

(3) $$\alpha_{ij}=\frac{exp\left(e_{ij}\right)}{\sum_{k\in N\left(i\right)}exp\left(e_{ik}\right)}$$

This normalisation ensures that the attention coefficients are comparable across the neighbourhood of node i. Assuming a single-layer feedforward neural network as the attention mechanism a, and employing the LeakyReLU (Leaky Rectified Linear Unit) activation function, which introduces a small non-zero response for negative inputs to maintain gradient flow and prevent inactive neurons, the attention coefficients can be calculated as follows:

(4) $$\alpha_{ij}=\frac{exp\left(LeakyReLU({\vec{a}}^{⊤}\left[x_{i}^{\prime }\|x_{j}^{\prime }\right])\right)}{\sum_{k\in N\left(i\right)}exp\left(LeakyReLU({\vec{a}}^{⊤}\left[x_{i}^{\prime }\|x_{k}^{\prime }\right])\right)}$$

in which $a^{⊤}$ denotes the transpose of a (the attention parameter vector), and ∥ indicates concatenation.

4. Feature aggregation: Each node’s output feature is computed as a weighted sum of its neighbours’ transformed features:

(5) $$x_{i}^{\prime \prime }=\sigma \left(\sum_{j\in N\left(i\right)}\alpha_{ij}x_{j}^{\prime }\right)$$

In this equation, σ denotes an activation function (e.g., ReLU) and $x_{i}^{\prime \prime }$ represents the refined feature for node i.

5. Multi-head attention: To improve stability and expressiveness, multiple attention mechanisms (heads) are employed. Each head independently computes its own set of attention coefficients and aggregated features:

(6) $$x_{i}^{\prime \prime }={\|}_{k=1}^{K}\sigma \left(\sum_{j\in N\left(i\right)}\alpha_{ij}^{\left(k\right)}x_{j}^{\prime }\right)$$

here, K denotes the number of attention heads; for the final layer, concatenation is replaced by averaging as follows:

(7) $$x_{i}^{\prime \prime }=\sigma \left(\frac{1}{K}\sum_{k=1}^{K}\sum_{j\in N\left(i\right)}\alpha_{ij}^{\left(k\right)}x_{j}^{\prime }\right)$$

**Figure 2:**
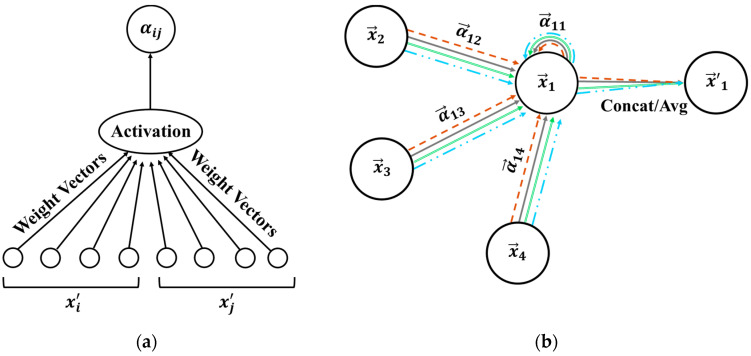
A schematic of the (**a**) self-attention mechanism and (**b**) multi-head attention mechanism in the GAT layer [39].

Figure 2a and Figure 2b present the attention mechanism described above, e.g., $e_{ij}$, and the multi-head attention when there are four heads, i.e., K=4, by node 1 and its neighbour nodes, respectively.

In Figure 2b, each line style and colour represent independent (here four) attention calculations.

### 2.3. Domain Adaptation

The DA stage of GAT-CAMDA combined DANN with MMD and CORAL loss functions to handle more severe distribution gaps between the source and target domains.

#### 2.3.1. Domain-Adversarial Neural Network

The DANN unit of GAT-CAMDA consists of a feature extraction unit that is responsible for projecting the source and target domain data into a shared space. Since in GAT-CAMDA an extra feature extraction stage is applied (i.e., GATs), the feature extractor unit of the DANN projects the distilled features from GATs to the shared space.

There are two heads connected to the DANN, i.e., a damage classifier and a domain discriminator. The damage classification unit predicts fault classes, while the domain discriminator determines whether input data come from the source or target domain.

Looking at Figure 1, it can be seen that a Gradient Reversal Layer (GRL) is used between the feature extractor and domain discriminator [40]; during the forward pass, the GRL acts as an identity layer, but during backpropagation, it reverses the gradients by −1.

**Figure 3:**
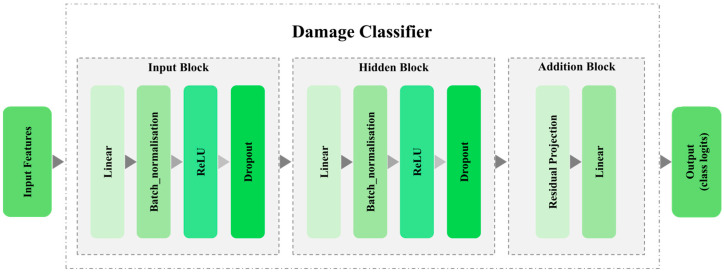
Schematic of damage classifier in GAT-CAMDA.

The damage classifier consists of three blocks, i.e., input, hidden, and addition blocks, each of which is composed of various layers such as linear layers, batch normalisation, and dropout, among others. Figure 3 presents a schematic of this neural network.

Structuring an overly complex damage classifier in DA tasks can lead to overfitting to the source domain [41]. Consequently, it is crucial to establish a trade-off between the model’s complexity and its capacity to generalise effectively to target domains. This balance ensures that the classifier maintains robust performance across diverse domains without being unduly influenced by the intricacies of the source data. As a result, a simple structure was chosen as the classifier in GAT-CAMDA.

In contrast, the domain discriminator comprises five blocks. Each block contains a linear layer followed by a LeakyReLU activation to introduce non-linearity while avoiding the dying ReLU problem. Dropout layers are included for regularisation, and the final block maps the learned features to domain-specific logits, enabling discrimination between source and target domains. A deeper discriminator architecture was selected to ensure sufficient representational power to capture subtle domain shifts and to generate a meaningful adversarial signal for the feature extractor during training. This design choice establishes an equilibrium between the discriminator and the classifier: the classifier remains generalisable, while the discriminator is strong enough to challenge it, thereby promoting robust and domain-invariant feature learning.

The loss functions for damage classifier ($L_{D}$) and domain discriminator ($L_{T}$) are well discussed in the literature [40].

#### 2.3.2. Maximum Mean Discrepancy

The MMD quantifies the distance between the average feature representations. This metric is pivotal in evaluating how effectively the feature distributions of the two domains align, which is essential for successful DA. The MMD ($L_{M}$) between two sets of observations belonging to domain s and t can be computed as follows [42]:

(8) $$L_{M}=\;{\left\|\frac{1}{n}\sum_{i=1}^{n}\emptyset \left({x^{\prime \prime }}_{s_{i}}\right)-\frac{1}{m}\sum_{j=1}^{m}\emptyset \left({x^{\prime \prime }}_{t_{j}}\right)\right\|}^{2}$$

in which ${x^{\prime \prime }}_{s_{i}}$ and ${x^{\prime \prime }}_{t_{j}}$ represent the sets of features from the source and target domains, respectively. The function ∅ maps these features into a higher-dimensional feature space, and n and m are the number of samples in each domain.

By mapping the features into a higher-dimensional space, MMD loss effectively captures more intricate patterns and discrepancies between the domains; a lower MMD loss indicates a greater similarity between the domains, which is indicative of improved DA performance.

When applying MMD, it is essential to select an appropriate kernel type, such as linear or Radial Basis Function (RBF). Specifically, for RBF kernels, adjusting the Gamma parameter is crucial, as it governs the kernel’s bandwidth and subsequently influences the sensitivity of the MMD measure to discrepancies in feature distributions.

#### 2.3.3. Correlation Alignment

The CORAL loss functions as a metric for evaluating the alignment between two domains by quantifying the discrepancies in their feature covariance matrices. Within the scope of DA, it is employed to align the data distributions of the source and target domains, thereby reducing statistical variations to enhance generalisation. By minimising this loss, the features that are learned become more consistent across both domains, thereby increasing the prediction accuracy for the target domain. The central concept of CORAL loss involves addressing domain shift by aligning second-order statistics, specifically through the minimisation of the Frobenius norm of the difference between the covariance matrices of the source and target domains. The CORAL loss ($L_{C}$) function can be expressed as follows [43]:

(9) $$L_{C}=\;\frac{1}{4d^{2}}{\left\|Cov\;{x^{\prime \prime }}_{s}-Cov\;{x^{\prime \prime }}_{t}\right\|}_{F}^{2}$$

where $Cov\;{x^{\prime \prime }}_{s}$ and $Cov\;{x^{\prime \prime }}_{t}$ denote the covariance matrices of the source and target features, respectively, d represents the dimensionality of the features, and ${\left\|\cdot \right\|}_{F}$ refers to the Frobenius norm. CORAL loss can be computed on a per-class basis or as an overall measure. Typically, the aggregate CORAL loss is more indicative, as it captures the general alignment across all classes, thereby ensuring that the adaptation generalises well across the entire domain rather than merely fitting specific classes.

### 2.4. Training Process of GAT-CAMDA

The total training loss comprises weighted losses from damage classification, domain discrimination, MMD, and CORAL, expressed as follows:

(10) $$L_{total}=\;\lambda_{1}\times L_{D}+\lambda_{2}\times L_{T}+\lambda_{3}\times L_{M}+\lambda_{4}\times L_{C}$$

where $\lambda_{1}$, $\lambda_{2}$, $\lambda_{3}$, and $\lambda_{4}$ are the weights assigned to the classification, discriminator, MMD, and CORAL losses, respectively.

In this hybrid DA framework, unlike the damage classification loss ($L_{D}$) and the domain discriminator loss ($L_{T}$), which each have explicit backpropagation paths due to their association with trainable network components, MMD ($L_{M}$) and CORAL ($L_{C}$) losses act as regularisation terms applied directly to shared feature representations. Consequently, their gradients are propagated only through the feature extractor to encourage domain-invariant feature learning, rather than through separate trainable branches.

Each training iteration comprises forward and backward propagation within a standard optimisation loop. During the forward pass, data from both domains are processed through the feature extractor to generate embeddings, which are subsequently fed into the damage classifier and the domain discriminator. The GRL ensures that these embeddings do not reveal their domain origin to the discriminator, thereby promoting domain-invariant feature learning. The MMD and CORAL losses are also computed once per iteration to minimise discrepancies between the feature distributions of the two domains.

During the backward pass, the combined losses update the model parameters as defined in Equation (10). This iterative process is fully automated and follows conventional neural network training procedures, without any manual adjustment or heuristic matching between the source and target domains. The parameter γ regulates the strength of the GRL by scaling the gradients associated with the domain discriminator loss $L_{T}$. Initially, γ is small to maintain training stability and is progressively increased according to a sigmoidal schedule defined as follows:

(11) $$\gamma =\gamma_{max}\left(\frac{2}{1+exp\left(-10p\right)}-1\right)$$

in which $p=\frac{Epoch}{{Epoch}_{max}}$, $\gamma_{max}$ is the maximum value of γ, and ${Epoch}_{max}$ is the total number of training epochs. This adaptive scheduling strategy enables gradual and effective TL while preserving the discriminative capacity of the extracted features for damage classification.

Designed as a semi-supervised framework, GAT-CAMDA does not use the labels from the target domain observations during the DA process. Additionally, given that there are multiple target domains, i.e., signals recorded at various temperatures, these are consolidated into a single target domain to further assess the generalisability of the framework. Algorithm 1 denotes a pseudocode for GAT-CAMDA.
**Algorithm 1.** GAT-CAMDA framework for SHM.**Input:** Source domain data and labels, target domain data, target domain labels (held out for the validation and testing). **Output:** Trained GAT-CAMDA model, feature-space alignment and sensor-importance visualisations, final classification performance on source test and target test. **1. Configuration and Setup** **1.1** Set the device to GPU if available. **1.2** Initialise random seeds for reproducibility. **1.3** Define global configuration (e.g., hidden dimensions, batch size). **2. Data Preprocessing** **2.1** Load source and target data. **2.2** Split source data into training, validation, and testing sets. **2.3** Split target data into training, validation, and testing sets, supporting stratification. **2.4** Convert labels to tensors and create graph-based representations of the data. **3. Model Initialisation** **3.1** Define the GNN-based feature extractor using GAT. **3.2** Define the discriminator for domain classification. **3.3** Define the classifier for damage classification. **3.4** Initialise the DANN. **4. Training the Model** **4.1** For each epoch:    **4.1.1** Compute the adaptive weight for domain-adversarial loss.    **4.1.2** For each batch of source and target data:       **a.** Forward pass through the feature extractor, classifier, and discriminator.       **b.** Compute classification loss, domain loss, MMD loss, and CORAL loss.       **c.** Backpropagate the combined loss and update model parameters. **4.2** Perform early stopping based on validation loss. **5. Hyperparameter Optimisation** **5.1** Use Optuna for hyperparameter tuning with a defined search space. **5.2** Optimise learning rate, weight decay, loss weights, and model architecture based on validation set of target domain. **5.3** Train and evaluate the final model with the best hyperparameters. **6. Model Evaluation** **6.1** Evaluate the model on the source test set for classification accuracy. **6.2** Evaluate the model on the target validation set for domain adaptation performance. **6.3** Generate confusion matrices and classification reports. **7. Feature-Space Visualisation** **7.1** Extract feature embeddings using the trained model. **7.2** Apply t-SNE for dimensionality reduction. **7.3** Visualise embeddings before and after domain alignment. **8. Sensor Importance Analysis** **8.1** Compute attention scores for each sensor from the GAT layers. **8.2** Normalise and visualise sensor importance scores. **9. Final Outputs** **9.1** Trained GAT-CAMDA model. **9.2** Visualisations of feature alignment and sensor importance.

### 2.5. Data Synthesising

As will be shown in the following section, the employed dataset contains observations for all health states at a baseline temperature ($T_{0}$, e.g., 30 °C), whilst for the remaining temperatures only healthy measurements are available. To address the lack of data at other temperatures and for the damaged plates, a data augmentation stage was applied. The methodology extrapolates the behaviour of damaged signals at new target temperatures ($T^{*}$) by leveraging the temperature dependence observed in healthy signals.

First, the data for the healthy and damaged plates were separated based on their labels, ensuring a clear delineation of reference (healthy) and target (damaged) conditions. The average healthy response $\bar{x}$($T_{i}$) was computed for each available temperature $T_{i}$; a spline interpolation function was then fitted to these mean signals, providing a continuous approximation of $\bar{x}$(T) across the temperature range. Using this interpolation, the mean healthy signal at the target temperature $\bar{x}$($T^{*}$) was estimated as follows:

(12) $$\bar{x}\left(T\right)=Interp\left(\left\{\bar{x}(T_{i})\right\},\left\{T_{i}\right\}\right)$$

To derive the temperature-specific scaling factor for the damaged data, the ratio between the mean healthy signals at the target and baseline temperatures was employed:

(13) $$S=\;\frac{\bar{x}(T^{*})}{\bar{x}(T_{0})}$$

This scaling factor S was then applied to the damaged signals at the baseline temperature,$x_{damaged}(T_{0})$, to generate the corresponding signals at the target temperature. A small stochastic term ϵ, drawn from a Gaussian distribution with an incredibly low variance, was included to introduce realistic variability. Thus, the synthesised damaged data were obtained as follows:

(14) $${\;x}_{damaged}\left(T^{*}\right)={\;x}_{damaged}(T_{0})\cdot S\cdot \left(1+\epsilon \right)$$

Following this procedure, new labels were assigned to the synthesised datasets to maintain consistency with the classification scheme.

### 2.6. Hyperparameter Optimisation

The optimisation process applies Optuna [44] to maximise the classification accuracy on the validation set of the target domain; this technique employs the Tree-structured Parzen Estimator (TPE) to model the probability distribution of J(θ) being maximised. This approach allows the optimiser to focus on hyperparameter regions that are more likely to enhance the classification accuracy. The TPE method effectively constructs a model of J and updates this model iteratively as new data (hyperparameter values and their corresponding $A_{t}$) are observed.

The objective function J is designed to maximise the classification accuracy of the validation subset of the target domain ($A_{t}$); this function can be formulated as follows:

(15) $$J\left(\theta \right)=\;A_{t}\left(\theta \right)$$

where θ and $A_{t}\left(\theta \right)$ present the vector of model hyperparameters and the classification accuracy on the target test set, respectively.

The goal of the optimisation is to find the optimal set of hyperparameters $\theta^{*}$ that maximises J:

(16) $$\theta^{*}=\;\begin{array}{c} argmax\;J\left(\theta \right)\\ \theta \end{array}$$

This optimisation problem involves dynamically adjusting θ to achieve the highest possible $A_{t}$, which indicates the model’s performance in effectively transferring learned knowledge from the source domain to the target.

### 2.7. Computing Sensor Importance

The computation of sensor importance in GAT-CAMDA directly relies on the attention coefficients $\left(\alpha_{ij}\right)$ defined in Section 2.2 and illustrated in Figure 2. These coefficients represent the learned weights that quantify the relative contribution of neighbouring nodes (sensors) to each node’s feature aggregation. For clarity, the symbol $\varsigma (\varsigma_{e}^{l})$ is subsequently used to denote the same attention weights when aggregated at the edge level in layer l, where each edge e∈E connects two sensors i and j.

To determine which sensor exerts the greatest influence on the final damage-detection outcome, the attention weights from the GAT are interpreted as indicators of each sensor’s contribution to the overall task. For each GAT layer, the attention weights corresponding to the edges connecting sensors are extracted and evenly distributed between the two connected nodes. These per-sensor contributions are then aggregated to obtain a batch-wise score, normalised within each batch, and subsequently accumulated and renormalised across all batches to yield a global sensor-importance metric.

(17) $$\begin{array}{c} S_{i}^{batch}+=\frac{\varsigma_{e}^{l}}{2}\\ S_{j}^{batch}+=\frac{\varsigma_{e}^{l}}{2}\\ S^{batch}⟵\frac{S^{batch}}{\sum_{i}S_{i}^{batch}}\\ S⟵\frac{S}{\sum_{i}S_{i}} \end{array}$$

In the following section, the calculated weights are visualised as a gradient bar chart for the three receiving sensors.

## 3. Case Study

A publicly available dataset, CONCEPT: CarbON-epoxy CompositE PlaTe, was employed to evaluate the effectiveness of the SHM framework designed in this study. The dataset includes Lamb wave measurements captured on a CFRP plate made of unidirectional plies, in both healthy and damaged states. The experiments focused on the effects of temperature variations and damage progression on the structural integrity of the plate.

**Figure 4:**
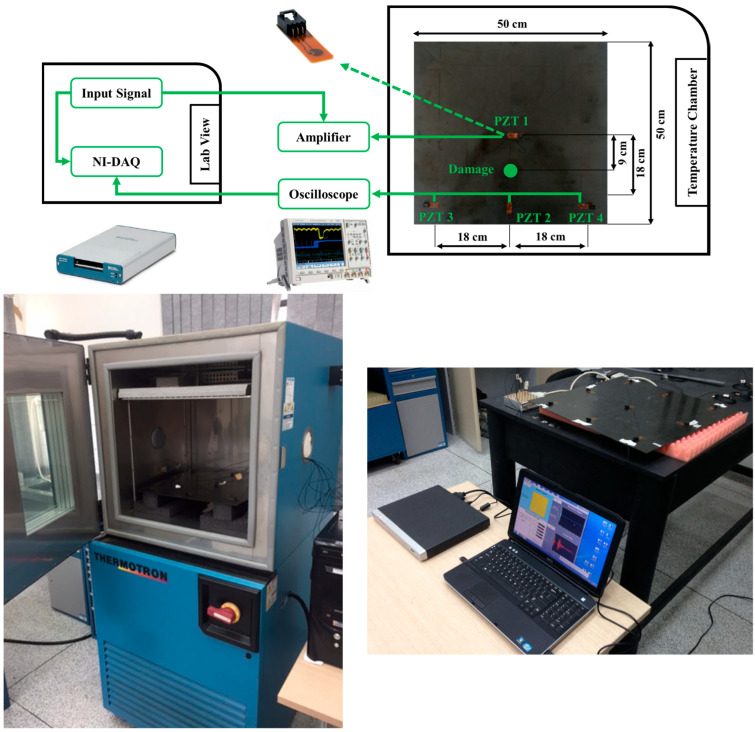
The test rig setup with CFRP plate and PZT transducers and temperature chamber from the CONCEPT experiments [45].

Four Lead Zirconate Titanate transducers from Acellent Technologies were bonded to the plate, with one (PZT1) serving as an actuator and the others (PZT2, PZT3, and PZT4) as receiving sensors. To minimise wave propagation constraints, the laminate was mounted under free–free boundary conditions. Figure 4 shows the setup along with the test rig and instrumentation; Table 1 presents the test rig and the instruments used.

The experiments were conducted in a Thermotron thermal chamber to precisely control temperature. The chamber uses an integrated cascade refrigeration system, with the 0 °C condition achieved through mechanical cooling rather than ambient freezing. A sinusoidal tone burst served as the excitation signal, with response signals sampled accordingly. Signal generation and measurement were managed by designated data acquisition systems, controlled via LabVIEW software. For the intact plate, 100 measurements were conducted at each of seven temperatures, ranging from 0 °C to 60 °C in 10 °C increments. For the damaged scenarios, 100 observations were recorded only at 30 °C, which was considered the baseline, with no measurements for damaged cases at other temperatures.

The damage scenarios were simulated by applying industrial adhesive putty to the composite plate’s surface, representing delamination-like defects. The damage was progressively increased in a localised region between PZT1 and PZT2 to examine the attenuation and propagation changes caused by the defects. Various health scenarios along with their severities and brief descriptions are summarised in Table 2, which also contains the labels allocated to the different temperatures. This dataset was employed in multiple research studies that have represented its capability in evaluating SHM frameworks in mitigating EOVs [15,46,47].

## 4. Result and Discussion

This section aims to show that the proposed SHM system, i.e., GAT-CAMDA, can accurately identify different health states of the structure, even with temperature changes that occur during operation. As previously mentioned, the source domain dataset comprises measurements from all health scenarios at a baseline temperature of 30 °C, with one PZT functioning as the actuator positioned at the centre of the plate (PZT1) and the remaining three PZTs (PZT2, PZT3, and PZT4) capturing the received Lamb waves. Initially, a data synthesis approach was employed as detailed in the preceding sections. Subsequently, the performance of the end-to-end hybrid DA and classification model was evaluated under the assumption that the testing data encompassed a range of background temperatures. Finally, a comparative study was performed to assess the superiority of GAT-CAMDA. Additionally, the analysis identified which receiver sensors were more significant and which hyperparameters had a substantial impact on the results.

### 4.1. Dataset Complementation

To produce data for target temperatures, e.g., 0 °C, 10 °C, 20 °C, 40 °C, 50 °C, and 60 °C, the interpolation and extrapolation processes were executed for each temperature and sensor using MATLAB (R2023a)^®^. The spline interpolation technique was employed to estimate signal values at unmeasured temperatures. In synthesising the damaged scenario data for new temperatures, a scaling factor, which was empirically derived from the healthy signals across different temperatures to reflect realistic temperature-dependent attenuation trends in guided wave propagation, was applied to adjust the signals. To add realism and account for natural variations in the data, a controlled variability of ±1% was introduced to the scaled signals. For each of the damaged health scenarios at the target temperatures, 100 observations were generated.

**Figure 5:**
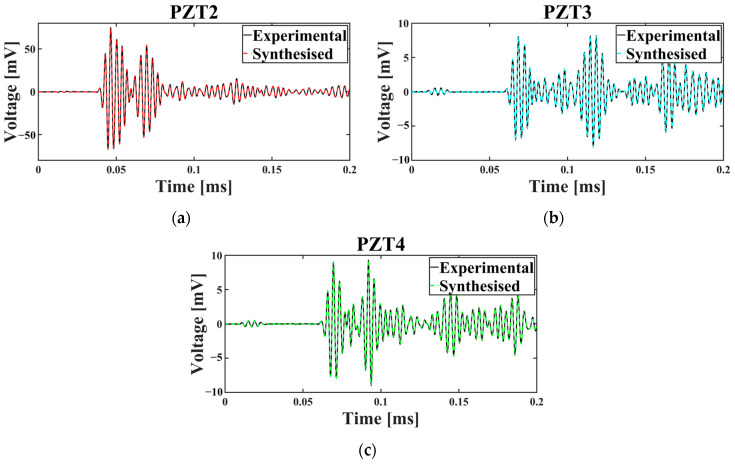
Time-domain signal of the experimental and synthesised data for the healthy plate for (**a**) PZT2, (**b**) PZT3, and (**c**) PZT4.

To evaluate the effectiveness of the applied data synthesis, the source temperature, e.g., 30 °C, was employed. To this end, among the 100 observations belonging to the healthy situation, 50 observations were selected randomly and employed to produce synthesised data, while the remaining 50 samples were used for the validation phase. Figure 5 displays an experimental observation of the intact plate, i.e., C0 (recorded at the baseline temperature) alongside its synthesised counterpart for the three channels.

Plots in Figure 5 show that the experimental and the synthesised data are close to each other for all three sensors in the time domain. To further analyse whether the synthesised data mimic the behaviour of the experimental signals, two metrics, namely Dynamic Time Warping (DTW) [48] and Cross-Correlation (CC) [49], were employed. Data was generated for the base temperature of 30 °C, which includes observations for all health scenarios. From each health state, 50 observations were used for data synthesising, while the remaining 50 observations per class were reserved for comparison with the synthesised data; this approach ensured a balanced and consistent evaluation process.

**Figure 6:**
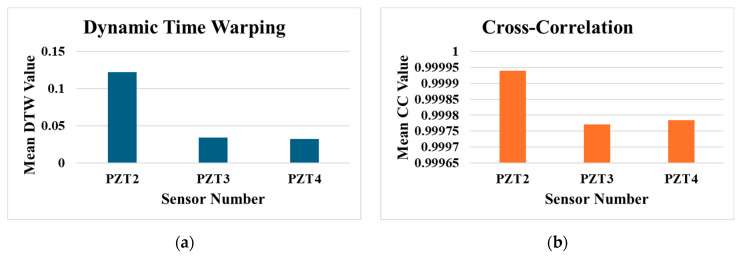
Comparison of synthesised and real data across sensors (PZT2, PZT3, PZT4) at 30 °C using (**a**) mean DTW values and (**b**) mean CC values.

DTW measured the optimal alignment between temporal sequences, with lower DTW values indicating greater similarity between the real and synthesised signals. CC assessed the linear relationship between the signals, where values close to −1 or +1 demonstrated higher similarity in amplitude and phase. The results of this validation process are presented in the bar graphs of Figure 6a,b, providing a visual representation of the synthesised data’s fidelity to the original observations by measuring the average values of DTW and CC metrics for the three sensors.

The acquired results demonstrate the effectiveness of the data synthesising process, as indicated by the low DTW values and CC values near 1 across all sensors. For PZT2, the DTW value is slightly higher (0.122) compared to PZT3 (0.034) and PZT4 (0.0321), which can be attributed to the larger amplitude of signals recorded by this sensor. However, the CC values for all sensors, e.g., PZT2 (0.99994), PZT3 (0.99977), and PZT4 (0.99979) are consistently close to 1, highlighting a strong similarity between the synthesised and real signals in terms of their temporal structure and amplitude relationships. These results validate the ability of the data synthesising process to generate realistic signals that align closely with the real observations, even in the presence of sensor-specific amplitude differences.

### 4.2. Damage Detection

To assess the performance of the proposed SHM framework, i.e., GAT-CAMDA, for detecting damage in composite plates subjected to EOVs, the data acquired at 30 °C was designated as the source domain. This source dataset was split into training (63%), validation (27%), and testing (10%). The remaining temperatures (0, 10, 20, 40, 50, and 60 °C) were combined to form a single dataset serving as the target domain, of which 70% was allocated to DA (with equal representation from each target temperature ensured by the ‘stratify’ option) and 15% was assigned to the validation set. The remaining 15% of the target dataset was reserved for testing; as a result, each of the 12 damage classes (C0–C11) in the target test set comprises 90 samples. Among these 90 observations, 15 belonged to 0 °C, 15 to 10 °C, and the others belonged to the remaining target temperatures. Table 3 provides a summary of the source and target datasets.

GAT-CAMDA was executed for 100 trials using an Optuna optimisation schedule to identify the framework’s optimal configuration, guided by 13 hyperparameters outlined in Table 4. The number of epochs was fixed at 50, and the objective function was defined as the maximum classification accuracy achieved on the target validation set.

During the DA phase, a random seed of 42 was then fixed to ensure reproducibility; the entire process of running GAT-CAMDA was performed through Jupyter Notebook in Python 3.11 (64-bit).

**Figure 7:**
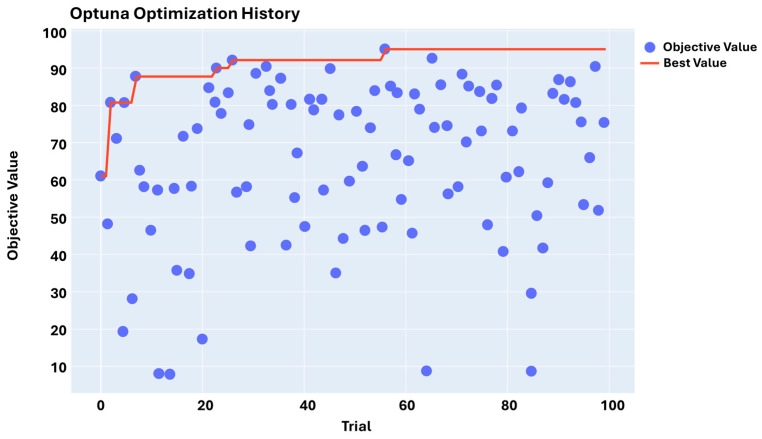
Hyperparameter optimisation history.

Executing GAT-CAMDA through the mentioned optimisation scheme, the highest damage detection accuracy was found at trial 56, as can be observed in the optimisation history plot (e.g., Figure 7).

Results from Figure 7 illustrate the extent to which choosing suitable hyperparameters can affect the outcome. For instance, at trial 20, the accuracy reached nearly 20%, while in the most optimal scenario (i.e., trial 56), it developed to 95.83%.

**Figure 8:**
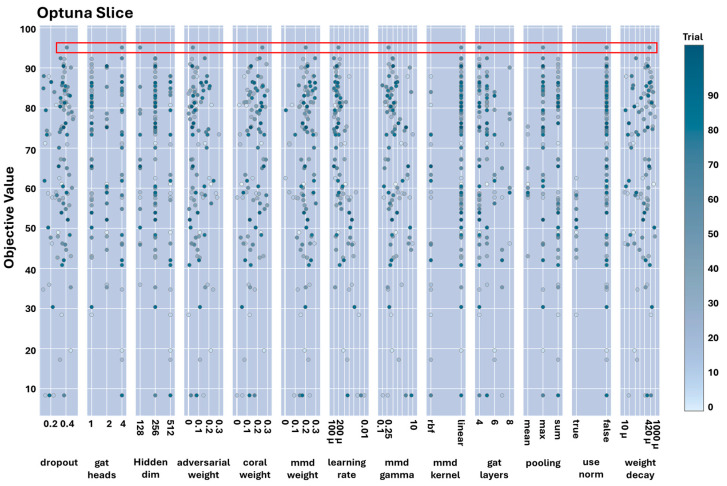
Slice plot of hyperparameters during optimisation.

The slice plot in Figure 8 shows the values and options (for the categorical hyperparameters such as pooling function, normalisation approach, and the type of MMD kernel) throughout the optimisation process and, more specifically, the trial in which the most optimal objective function was discovered; the hyperparameter values for this trial are surrounded by the red rectangle.

The prominent results presented in Figure 8 and more specifically red box indicate that implementing a normalisation process within GAT-CAMDA led to inferior outcomes compared to using the original signals during the signal processing stage. Additionally, employing lower learning rate values resulted in improved performance. Furthermore, the mean pooling function was found to be unsuitable for GAT-CAMDA, as the highest accuracy achieved in this scenario was approximately 75%.

**Figure 9:**
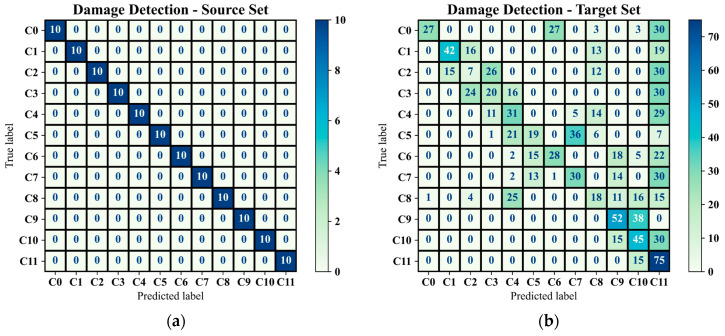
Classification outcomes through GAT-CAMDA without DA section for (**a**) source domain test set and (**b**) target test set.

Before representation of the damage detection results utilising the full version of GAT-CAMDA and to demonstrate the effectiveness of the feature extraction phase and the capability of the classifier, the trained GAT-CAMDA framework was evaluated using the test subset of the source domain. Moreover, the DA component of GAT-CAMDA was removed, and the trained network was subsequently tested on the target test subset. The confusion matrices presented in Figure 9a,b display the damage detection outcomes for both of these scenarios, respectively.

The confusion matrix presented in Figure 9a demonstrates that the implemented feature extraction method can effectively differentiate between the various damage scenarios. Furthermore, these results confirm that the classifier is trained impeccably and has a high capability in the classification phase. Figure 9b, on the contrary, reveals that this well-trained network fails to accurately identify the different damage classes (approximately 36% accuracy) when the feature distribution space of the test set is altered due to EOVs. Consequently, these findings underscore the need for a DA phase to ensure that the framework trained on the source domain functions properly when applied to data from the target domain.

**Figure 10:**
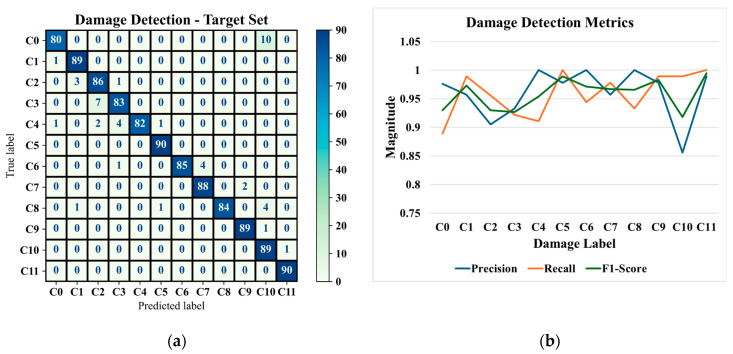
Classification outcomes produced by the optimum GAT-CAMDA framework on the target test set, showing (**a**) the confusion matrix and (**b**) the classification performance metrics.

The outcomes of the most optimal framework in classifying the target test set are depicted in Figure 10a,b; the latter shows the classification metrics, i.e., Precision, Recall, and F1-Score [50].

The classification metrics indicate that the proposed GAT-CAMDA framework achieves a robust differentiation among the 12 structural health scenarios, maintaining high precision and recall values across most classes (with an average accuracy of 95.83%). In particular, classes exhibiting a precision of 1 reveal that the model consistently classifies those specific damage conditions without false positives, demonstrating its capacity to accurately identify even subtle differences in wave propagation patterns. Moreover, the consistently elevated recall across all classes signifies a low rate of missed damage instances, a critical requirement in damage detection tasks where undetected flaws may compromise structural integrity. The marginally reduced precision for C10 nonetheless retains a high recall, suggesting that, although the model occasionally misassigns instances into this category, it rarely overlooks genuine damage of that type.

**Figure 11:**
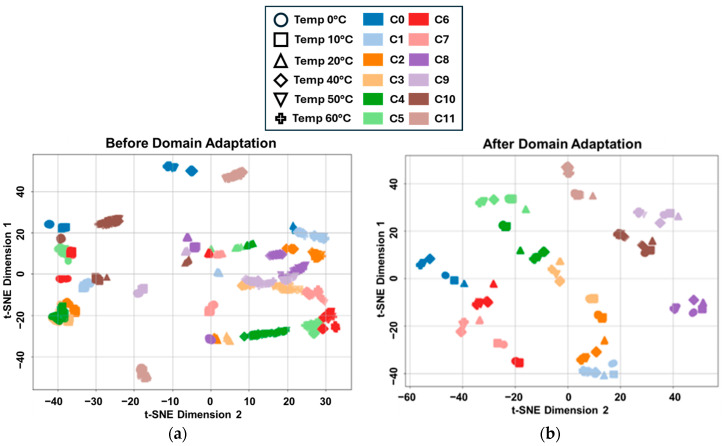
Two-dimensional t-SNE projections illustrating the distribution of extracted features (**a**) before DA and (**b**) after DA using the GAT-CAMDA framework.

To visualise the impact of GAT-CAMDA on feature distribution, t-distributed Stochastic Neighbour Embedding (t-SNE) [51] was employed. This dimensionality reduction technique projected the extracted features into a two-dimensional space, both before and after the hybrid DA stage within GAT-CAMDA. The resulting data distributions are displayed in Figure 11a and Figure 11b, respectively, with each scatter point representing an observation belonging to a specific temperature and damage scenario.

When comparing the t-SNE scatter plots before and after the GAT-CAMDA-based DA, a marked improvement in feature-space alignment is evident. Specifically, observations corresponding to identical temperature-damage scenarios become more cohesively clustered post-adaptation, illustrating that GAT-CAMDA effectively compensates for the distributional shifts introduced by varying temperature. Consequently, data points representing identical classes converge into denser, well-separated regions, underscoring the framework’s ability to disentangle and preserve class-discriminative characteristics. These findings underscore GAT-CAMDA’s capacity to capture subtle signal features and thereby distinguish healthy from damaged states with high fidelity, which is an essential requirement for a robust and generalised SHM system. By mitigating the confounding effects of temperature variation, GAT-CAMDA enhances inter-class separability while maintaining intra-class consistency, providing a scalable and reliable platform for advanced SHM applications.

### 4.3. Comparative Study

The performance of the proposed GAT-CAMDA framework was compared with multiple established feature-based DA methods. All models used an equal number of observations, 70% of the combined target domain, for the feature alignment phase. The hyperparameters of these baseline models were tuned by trial and error to determine their best performance. In total, 10 DA methods, i.e., TCA, Feature Selection with MMD (fMMD), CORAL, Deep CORAL, SrcOnly Prediction (PRED), Subspace Alignment (SA), Domain-Adversarial Training of Neural Networks (DANN), DUA (Dynamic Unsupervised Adaptation), and BAR (Balanced Adaptation Regularisation-based Transfer Learning) were evaluated using the augmented version of the CONCEPT dataset. Among these, two methods are supervised, while the remaining eight are UDA approaches.

Optuna-based optimisation was not employed for the baseline models to maintain consistency with their originally reported configurations and to avoid introducing optimisation biases unrelated to their native designs. Similarly, GAT-based feature extraction was not applied to these methods, as doing so would alter their underlying architectures and compromise the validity of the comparison. Table 5 presents the classification metrics for all approaches alongside the proposed GAT-CAMDA framework.

**Figure 12:**
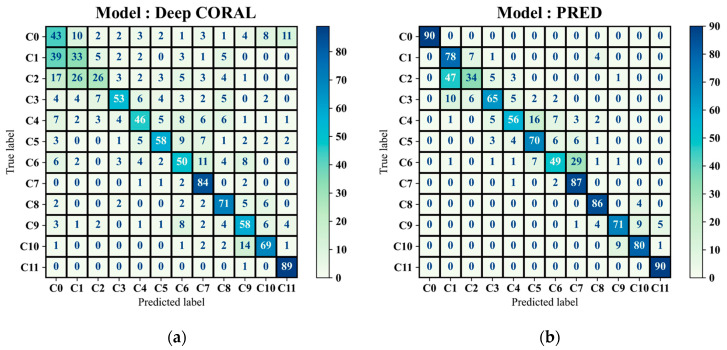

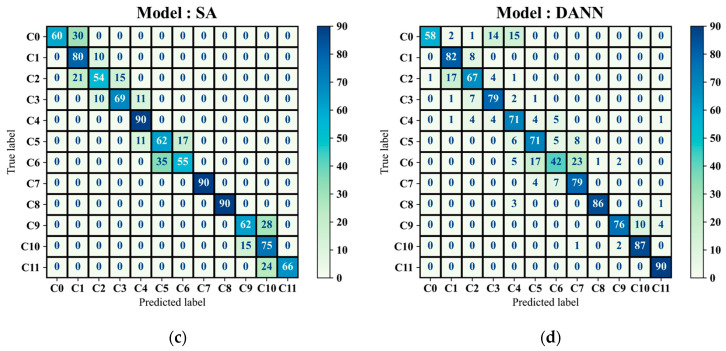
Damage detection result utilising (**a**) Deep CORAL, (**b**) PRED, (**c**) SA, and (**d**) DANN.

The results in Table 5 show that, although it is a challenging case study because of the integration of disjoint target domains, GAT-CAMDA sharply outperformed almost all of the comparative approaches. It achieved an accuracy of 95.83%, with precision and F1-scores each at 0.96. This is further improved compared to BAR, which, despite outperforming the other methods assessed, with an accuracy of 91.02%, precision and F1-scores of 0.92 and 0.92, respectively, was not as effective as GAT-CAMDA. The better performance underlines the robustness of the procedure proposed within this work. Confusion matrices in Figure 12a to Figure 12d display the classification results of Deep CORAL, PRED, SA, and DANN, respectively, on the target test set, providing further visual insight into the class-wise performance.

From Figure 12, it is evident that GAT-CAMDA exhibits exceptional precision in classifying the intact plate scenario (C0) and distinct damage states (C5 and C11) compared to other methods such as SA and DANN, which frequently misclassify closely related classes (e.g., C2 and C3). However, it is important to acknowledge that C6 presented considerable classification challenges for all evaluated methods, although GAT-CAMDA managed to resolve this challenge more effectively.

### 4.4. Hyperparameter Importance

**Figure 13:**
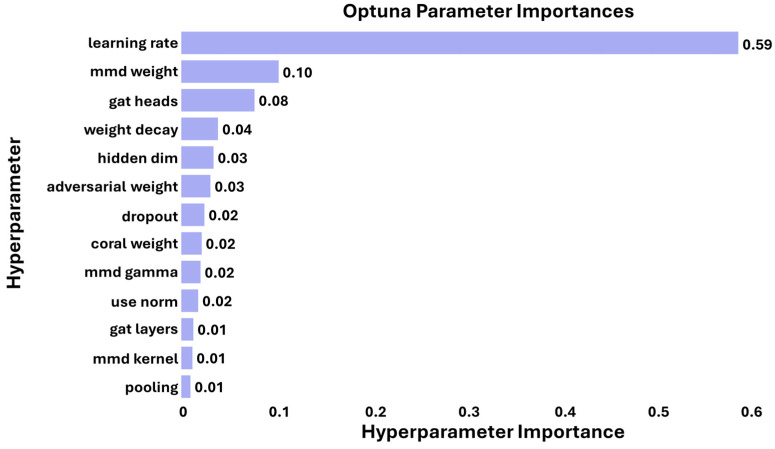
The relative influence of hyperparameters on model performance in damage detection.

To illustrate the relative influence of the 13 hyperparameters involved in the optimisation process on the outcomes of GAT-CAMDA, their importances were calculated using Optuna and are depicted in Figure 13. This visual representation facilitates informed decision-making by highlighting the hyperparameters with the most significant impact, allowing future researchers to prioritise these for fine-tuning to enhance the model’s accuracy and efficiency in future applications.

From Figure 13, it can be observed that the learning rate is the most critical hyperparameter, with a significant importance value of 0.59, underlining its pivotal influence on model performance. The weight of the MMD loss and the number of heads in the GAT also hold considerable importance, with values of 0.10 and 0.08, respectively, highlighting their substantial roles in shaping the model’s effectiveness. Additionally, the dimension of the hidden layer, though less influential at 0.04, is crucial for fine-tuning the model’s operational capabilities in damage detection. This analysis underscores the necessity of prioritising these hyperparameters during the optimisation process to maximise the model’s accuracy and efficiency.

It is important to note that the hyperparameter importance scores in Figure 13 represent global aggregated effects estimated by Optuna’s TPE across all 100 optimisation trials, rather than performance trends observed in individual configurations such as those shown in Figure 8. Consequently, parameters exhibiting consistent influence across multiple trials, such as the learning rate, receive higher importance values, even if certain categorical settings (e.g., normalisation = true or specific MMD kernels) occasionally produce pronounced local variations in accuracy. This explains why the learning rate dominates the global importance ranking, whereas other factors such as the MMD kernel, pooling type, and normalisation show significant but less consistent effects. The two figures are therefore complementary: Figure 8 visualises trial-level sensitivity, while Figure 13 summarises overall influence across the entire optimisation landscape.

### 4.5. Sensor Importance

**Figure 14:**
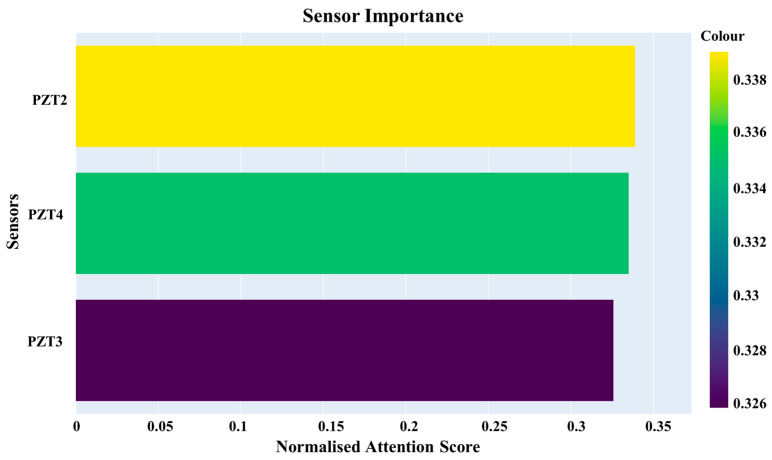
Sensor contribution levels determined by attention mechanism for the normalised signals.

In the GAT-CAMDA framework, the attention scores for each sensor were normalised to effectively evaluate their significance in the damage detection process. These scores were determined by the extent to which each sensor contributed to identifying structural anomalies, with all calculations having been performed under the optimal configuration settings of the framework. The normalised scores ranged from 0 to 1, providing a consistent and comparative metric of sensor importance. Figure 14 displays these scores, visualised as a gradient bar chart, which illustrates the relative contribution of each sensor to the overall damage detection capability.

As previously noted, the most optimal framework identified was for scenarios in which the signals were not normalised. Figure 5 demonstrates that the amplitude of the signals recorded by PZT2 is approximately eight times greater than those from the other two receivers, PZT3 and PZT4. Furthermore, the signal amplitude captured by PZT4 is marginally higher than that of PZT3. These differences in signal amplitude play a pivotal role in determining the final outcome, as evidenced by the calculated sensor importance scores, whereby PZT2, PZT4, and PZT3 exhibit progressively higher importance in the final output, respectively.

**Figure 15:**
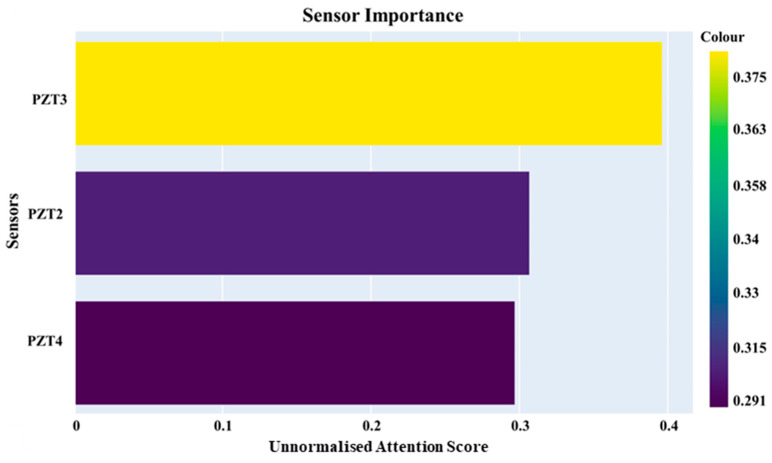
Sensor contribution levels determined by attention mechanism for unnormalised signals.

The same optimal framework was assessed under the assumption that normalisation was not applied, while maintaining the other hyperparameter values unchanged; the computed sensor importances are displayed in Figure 15.

The pronounced significance of PZT3 within the designed SHM framework on the assumption that the unnormalised versions of the data were fed into the signal processing and learning network is consistent with the findings in [47]. It was confirmed that PZT3 exhibits a wider confidence interval in its signal data compared to PZT2, indicating greater variability. This increased variability is influenced by temperature changes, which affect the material’s mechanical properties. Specifically, as temperature rises, the stiffness of the CFRP laminate decreases, with the shear modulus $G_{12}$ reducing by 7%. This decline is much higher than the 1.3% decrease observed in the elastic modulus $E_{1}$. This difference is crucial because PZT3 is positioned at a 45-degree angle from the excitation point, making it particularly sensitive to changes in the shear modulus due to its orientation relative to the main direction of wave propagation. The heightened signal dispersion at PZT3 results from its unique placement and the viscoelastic characteristics of the epoxy resin, which are not present in the carbon fibres [47]. Consequently, despite a lower signal-to-noise ratio, PZT3 shows an enhanced ability to detect subtle anomalies in wave propagation that may indicate structural damage. Figure 15 reflects the intricate relationship between sensor placement, material properties, and wave behaviour in composite structures.

## 5. Conclusions and Future Work

The GAT-CAMDA framework was designed to address the challenge of variability in SHM caused by temperature fluctuations, with its effectiveness demonstrated on a CFRP plate. Leveraging GATs for advanced feature extraction and incorporating MMD, CORAL, and adversarial losses for discriminative DA, the framework successfully aligned feature distributions across diverse temperature domains. This alignment significantly improved the accuracy of damage detection within the system. Comparative studies with established methods further validated the framework’s superior performance, highlighting its ability to handle the complexities of temperature-integrated target domain datasets.

To address the challenge of data scarcity, particularly for unmeasured damage conditions at different temperatures, a synthetic data generation algorithm was employed. This algorithm enriched the dataset by creating synthetic samples, helping the validation of the framework’s generalisability and robustness. As a result, the framework was extensively evaluated and refined for conditions that were not physically measured, ensuring consistent performance across a theoretical range of operational scenarios.

Explainability techniques played a crucial role in clarifying the decision-making processes of GAT-CAMDA. These techniques visualised the contributions of various sensors, identifying the most influential one for damage detection. Furthermore, the study displayed the impact of key hyperparameters on damage detection accuracy, providing valuable insights into optimal model configurations.

Although the GAT-CAMDA framework effectively addresses variability in SHM caused by temperature changes, further improvements are needed to enhance its computational efficiency for real-time deployment. This includes optimising graph processing algorithms and integrating hardware accelerations, such as Tensor Processing Units, to reduce processing times. Additionally, increasing the realism and diversity of synthetic data through advanced generative models could further strengthen the framework’s practical utility. As the current experiments were conducted using synthetic data, future work should include comprehensive validation on real-world experimental datasets to confirm the framework’s robustness and generalisability under practical operating conditions. Extending the framework’s adaptability to include a wider range of environmental conditions beyond temperature, such as humidity or load variations, could broaden its applicability across different industrial settings.

## Figures and Tables

**Table 1:** Summary of the test rig and instruments used in the CONCEPT experiments.

Category	Parameter	Value
Laminate plate	Dimensions (L × W × T)	500 mm × 500 mm × 2 mm
	Number of Plies	10
Transducers	Type	PZT
	Diameter	6.35 mm
	Configuration	One actuator, three sensors
Mounting conditions	Boundary condition	Free–Free
Temperature control	Range	0 °C to 60 °C
	Increment	10 °C
Excitation signal	Type	Five-cycle sinusoidal tone burst
	Frequency	250 kHz
Data sampling	Sampling rate	5 MHz
	Duration per measurement	100 ms
Data acquisition	Generation system	NI USB 6353
	Measurement system	Keysight DSO7034B
	Control software	LabVIEW

**Table 2:** Damage scenarios and severities for simulated defects in the CONCEPT experiment.

Damage Scenario	Severity (Area Covered)	Class Label	Description	Temperature (Degree Celsius)	Temperature Label
Healthy	0%	C0	No damage	0	0
				10	1
				20	2
				30	3
				40	4
				50	5
				60	6
Damaged D1	0.196%	C1	Industrial putty	30	3
Damaged D2	0.282%	C2	Increased coverage of putty		
Damaged D3	0.384%	C3	Further increase in coverage		
Damaged D4	0.502%	C4	Progressive increase		
Damaged D5	0.785%	C5	Larger area covered		
Damaged D6	1.13%	C6	Substantial coverage		
Damaged D7	1.53%	C7	Continued increase		
Damaged D8	1.95%	C8	Different progression pattern		
Damaged D9	2.01%	C9	Extensive coverage		
Damaged D10	2.27%	C10	High severity		
Damaged D11	2.54%	C11	Maximum simulated severity		

**Table 3:** Splitting the dataset for source and target domains.

Domain	Number of Observations per Class		
	Training	Validation	Testing
Source	63	27	10
Target	420	90	90

**Table 4:** Hyperparameter values for executing GAT-CAMDA.

Hyperparameter	Value	Hyperparameter	Value
Learning rate	$(1\times 10^{-4}\;$$,\;1\times 10^{-2}$)	Number of GAT heads	1, 2, 4
Weight decay	$(1\times 10^{-5}\;$$,\;1\times 10^{-3}$)	Pooling option	Max, Mean, Sum
Adversarial weight	(0, 0.3)	Normalisation	True, False
MMD weight	(0, 0.3)	MMD kernel	Linear, RBF
CORAL weight	(0, 0.3)	Gamma parameter	(0.1, 10)
Hidden dimension	128, 256, 512	Number of GAT layers	4, 8
Dropout rate	(0.1, 0.5)	Batch size	32

**Table 5:** Comparison of GAT-CAMDA with other feature-based methods.

Model	Accuracy (%)	Precision	F1-Score
TCA [52]	52	0.63	0.48
fMMD [53]	58	0.67	0.54
CORAL [43]	59	0.66	0.56
Deep CORAL [54]	63	0.63	0.62
PRED [55]	79	0.80	0.79
SA [56]	79	0.81	0.79
DUA [57]	82	0.81	0.79
DANN [40]	82	0.83	0.82
BAR [58]	91.02	0.92	0.92
GAT-CAMDA	95.83	0.96	0.96

## Data Availability

The employed dataset is available upon request via https://github.com/shm-unesp/DATASET_PLATEUN01?tab=readme-ov-file (accessed on 20 December 2024).

## Abbreviations

The following abbreviations are used in this manuscript:

BAR	Balanced Adaptation Regularisation-based Transfer Learning
CC	Cross-Correlation
CFRP	Carbon Fibre-Reinforced Polymer
CORAL	Correlation Alignment
DANN	Domain-Adversarial Training of Neural Networks
DA	Domain Adaptation
DTW	Dynamic Time Warping
DUA	Dynamic Unsupervised Adaptation
EOVs	Environmental and Operational Variabilities
FE	Finite Element
fMMD	Feature Selection with MMD
GATs	Graph Attention Networks
GRL	Gradient Reversal Layer
ML	Machine Learning
MMD	Maximum Mean Discrepancy
PRED	SrcOnly Prediction
PZT	Lead Zirconate Titanate
RBF	Radial Basis Function
ReLU	Rectified Linear Unit
SA	Subspace Alignment
SHM	Structural Health Monitoring
TCA	Transfer Component Analysis
TL	Transfer learning
t-SNE	t-distributed Stochastic Neighbour Embedding
TPE	Tree-structured Parzen Estimator
UDA	Unsupervised Domain Adaptation
